# Association of Dog and Cat Ownership with Incident Frailty among Community-Dwelling Elderly Japanese

**DOI:** 10.1038/s41598-019-54955-9

**Published:** 2019-12-09

**Authors:** Yu Taniguchi, Satoshi Seino, Mariko Nishi, Yui Tomine, Izumi Tanaka, Yuri Yokoyama, Tomoko Ikeuchi, Akihiko Kitamura, Shoji Shinkai

**Affiliations:** 1 Research Team for Social Participation and Community Health, Tokyo Metropolitan Institute of Gerontology, Tokyo, Japan; 2 Japan Environment and Children’s Study Programme Office, National Institute for Environmental Studies, Ibaraki, Japan

**Keywords:** Medical research, Risk factors

## Abstract

Accumulating evidence from studies of human–animal interaction highlights the physiological, psychological, and social benefits for older owners of dogs and cats. This longitudinal study examined whether experience of dog/cat ownership protects against incident frailty in a population of community-dwelling older Japanese. Among 7881 non-frail community-dwelling adults aged 65 years or older who completed a mail survey in 2016, 6,197 (mean [SD] age, 73.6 [5.3] years; 53.6% women) were reevaluated in a 2018 follow-up survey. Frailty was assessed with the Kaigo-Yobo Checklist. Incident frailty was defined as a score of four or higher in the follow-up survey. Overall, 870 (14.0%) were current dog/cat owners, 1878 (30.3%) were past owners and 3449 (55.7%) were never owners. During the 2-year follow-up period, 918 (14.8%) developed incident frailty. Mixed-effects logistic regression models showed that the odds ratio for incident frailty among dog/cat owners, as compared with never owners, current owners were 0.87 (95% confidence interval [CI]: 0.69–1.09) and past owner were 0.84 (0.71–0.98), after controlling for important confounders at baseline. In stratified analysis, the risk of incident frailty was lower for past dog owners than for cat owners. Longer experience of caring for a dog requires physical activity and increases time outdoors spent dog walking and thus may have an important role in maintaining physical and social function and reducing frailty risk among older adults.

## Introduction

Accumulating evidence from studies of human–animal interaction (HAI) shows benefits for health outcomes among pet owners of various ages^1–15^. Among older adults, dog/cat ownership was associated with greater physical activity in Western countries^11–13^, and our previous study of community-dwelling older Japanese showed that dog owners had greater physical function, and that dog and cat owners had better social function, than did never owners, even after adjustment for important sociodemographic and health characteristics^16^. These findings suggest that dog/cat ownership may have key role for older adults to maintain physically and socially active lifestyle.

Frailty is a growing public health concern worldwide over the last few decades. Physical frailty is a state in which cumulative aging-related deficits decrease reserve across multiple physiological systems^17,18^ and makes individuals more vulnerable to internal and external stressors and increases risks of adverse health outcomes, such as falls^19^, fracture^20^, disability^21^, hospitalization^22^, institutionalization^23^, dementia^18^, and premature death^24,25^. Although mechanism for frailty development remains unclear, social frailty (i.e. going out less frequency, rarely visiting friends, feeling less like helping friends or family, living alone and not talking to anyone all day) may precede and lead to the development of physical frailty^26^. We hypothesized that experience of dog/cat ownership might reduce the risk of incident frailty in later life through physically and socially active lifestyle. However, there are no data at present to confirm such a hypothesis. Furthermore, previous studies reported negative effect for dog/cat owners, for example, current and past dog/cat owners were more likely to have experienced a fall and hospitalized during the past year^16^ because of their higher physical activity levels. Some pet owners may suffer pet loss^11^, allergy, and zoonosis.

To increase healthy life expectancy, there is a growing interest in the effects of dog/cat ownership among older adults. This longitudinal study examined whether past or present dog/cat ownership is a protective factor for incident frailty, after controlling for important confounders, in a large sample of community-dwelling older Japanese. It is important to examine the association of dog/cat ownership with subsequent frailty risks among older adults, because some of past or present dog/cat owners may have experienced disadvantages by their pats. Japan has one of the world’s highest life expectancy and is also a super-aged society. The proportion of older adults is the highest in the world, and the number of pets exceed the number of children under the age of 15 with the nation’s declining birth rate. This study of the effects of dog/cat ownership on frailty among older Japanese is likely to yield insights regarding health promotion strategies in super-aging societies worldwide.

## Results

### Health characteristics

Mean (SD) participant age was 73.6 (5.3) years, 53.6% were women, and 19.1% were living alone. Overall, 870 (14.0%) were current dog/cat owners, 1878 (30.3%) were past owners and 3449 (55.7%) were never owners. Stratified by dog and cat ownership, 554 (8.9%) were current dog owners, 1466 (23.7%) were past dog owners and 4177 (67.4%) were never dog owners. 386 (6.2%) were current cat owners, 693 (11.2%) were past cat owners and 5118 (82.6%) were never cat owners.

Table 1 shows a comparison of baseline demographic and comprehensive health characteristics with experience of dog/cat ownership in 2016. Older adults with current dog/cat ownership were more likely to be younger, living with someone, and married, to have more education and higher equivalent incomes, to have fallen during the past year, and to have a smoking habit, higher motor fitness scale scores, closer interaction with neighbors, and higher frequency of going outdoors.

**Table 1:** Baseline Demographic and Health Characteristics of 6197 Community-Dwelling Older Japanese With and Without Experience of Dog/Cat Ownership.

Variable	Experience of dog/cat ownership			P-Value
	Current (n = 870, 14.0%)	Past (n = 1878, 30.3%)	Never (n = 3449, 55.7%)	
Sex (female)	51.4	55.1	53.4	0.186
Age, years (%)				<0.001
65–74	65.2	51.4	50.4	
75–84	34.8	48.6	49.6	
Household size (%)				<0.001
Living alone	8.9	17.5	22.0	
Living together	90.0	80.8	76.7	
Missing	1.1	1.7	1.3	
Marital status (%)				<0.001
Married	77.1	70.8	66.1	
Divorced, Widowed, Single	22.0	27.7	33.1	
Missing	0.9	1.5	0.9	
Educational attainment (%)				<0.001
Elementary school, Middle school, Others	17.6	17.6	24.3	
High school	36.9	37.6	40.4	
College, university, or graduate school	45.2	44.1	34.2	
Missing	0.3	0.6	1.0	
Equivalent income (%)				<0.001
<1,000,000 yen	4.1	4.5	5.2	
1,000,000 yen - 2,500,000 yen	26.8	27.2	31.6	
2,500,000 yen - 4,000,000 yen	24.3	26.2	28.9	
≥4,000,000 yen	28.5	25.9	18.3	
Unknown	16.3	16.3	16.0	
History of hypertension (%)				0.404
Yes	50.1	50.1	51.2	
No	47.0	47.3	45.4	
Missing	2.9	2.6	3.4	
History of hyperlipidemia (%)				0.588
Yes	43.1	41.1	41.2	
No	52.0	54.8	54.1	
Missing	4.9	4.2	4.8	
History of heart disease (%)				0.286
Yes	17.0	19.8	18.9	
No	77.6	76.1	76.7	
Missing	5.4	4.1	4.3	
History of stroke (%)				0.550
Yes	5.2	5.8	5.0	
No	89.5	90.0	90.7	
Missing	5.2	4.3	4.3	
History of diabetes mellitus (%)				0.924
Yes	16.3	15.3	15.3	
No	79.8	80.6	81.0	
Missing	3.9	4.0	3.8	
History of lung respiratory disease (%)				0.771
Yes	12.1	13.0	11.9	
No	83.2	82.8	83.9	
Missing	4.7	4.2	4.2	
Hospitalization during past year (%)	9.1	8.4	7.9	0.483
Fall during past year (%)	8.9	10.1	8.1	0.049
Alcohol drinking status (%)				0.345
Current	59.0	58.7	57.7	
Past	4.8	6.3	5.7	
Never	35.7	34.5	35.8	
Missing	0.5	0.4	0.8	
Smoking status (%)				<0.001
Current	14.6	9.8	9.9	
Past	33.8	34.0	29.6	
Never	50.9	55.6	59.7	
Missing	0.7	0.6	0.8	
Food variety (%)				0.520
≥4 points	42.0	42.8	41.9	
0–3 points	53.7	51.5	52.4	
Missing	4.4	5.6	5.7	
BMI (kg/m^2^)	22.9 (3.1)	22.6 (3.0)	22.7 (3.0)	0.079
Motor fitness scale (score)	11.8 (2.3)	11.7 (2.4)	11.4 (2.6)	<0.001
Exercise habit (%)				0.544
Yes	82.3	80.3	80.0	
No	17.2	19.0	19.2	
Missing	0.5	0.7	0.8	
Interaction with neighbors (%)				0.003
Significant relationship	26.4	28.4	26.4	
Conversation	44.3	40.4	38.3	
Exchange of greetings only, No social contact	29.2	31.2	35.3	
Missing	0.1	0.1	0.1	
Frequency of going outdoors (%)				<0.001
At least once a day	86.7	80.0	82.3	
Less than once every 2–3 days	13.3	20.0	17.7	
Self-rated health (%)				0.538
Excellent to good	85.2	86.8	85.3	
Fair to poor	10.0	8.4	9.6	
Missing	4.8	4.7	5.1	
GDS-5 (%)				0.361
≥2 points	26.1	23.8	24.3	
0–1 point	71.1	72.5	71.7	
Missing	2.8	3.7	4.0	

(SD). P values were calculated with the chi-square test or t-test. BMI, body mass index. GDS, Geriatric Depression Scale.

### Dog/cat ownership with incident frailty

During the 2-year follow-up, 918 (14.8%) out of 6197 participants developed incident frailty. Among dog/cat ownership, 13.2% of current owners, 13.4% of past owners and 16.0% of never owners developed incident frailty (chi-square test, *P* = 0.015). Corresponding values in current, past and never dog owners were 12.5%, 13.2% and 15.7% (chi-square test, *P* = 0.016), and corresponding values of cat owners were 14.2%, 13.7% and 15.0% (chi-square test, *P* = 0.632).

The associations of dog/cat ownership with incident frailty are shown in Table 2. As compared with never dog/cat owners, current dog/cat owners had 0.90 (95% confidence interval [CI]: 0.72–1.13) times and past owners had 0.85 (0.71–0.99) times as likely to develop incident frailty after controlling for sex and age at baseline. Even after adjustment for comprehensive sociodemographic and health characteristics at baseline, past dog/cat ownership was associated with a significantly lower odds ratio (OR) of incident frailty, 0.84 (0.71–0.98), and current ownership showed no significance (*P* = 0.237) but lower OR of 0.87. Estimated values (SE) for incident frailty by mixed-effects logistic regression models were −0.17 (0.08) in past dog/cat owners and −0.10 (0.11) in current owners, as compared with never owners.

**Table 2:** Independent Associations between Experience of Dog/Cat Ownership and Incident Frailty Among Community-Dwelling Older Japanese.

Independent Variable	Sex and age-adjusted OR (95% CI)	Multivariate-adjusted Model OR (95% CI)	Sex, age, physical function and physical activity adjusted OR (95% CI)	Sex, age, and social function adjusted OR (95% CI)
**Experience of dog/cat ownership**				
Never § (n = 3449, 55.7%)	1	1	1	1
Past (n = 1878, 30.3%)	0.85 (0.71–0.99)*	0.84 (0.71–0.98)*	0.84 (0.70–1.01)	0.88 (0.75–1.04)
Current (n = 870, 14.0%)	0.90 (0.72–1.13)	0.87 (0.69–1.09)	0.87 (0.69–1.10)	0.95 (0.75–1.19)
**Experience of dog ownership**				
Never § (n = 4177, 67.4%)	1	1	1	1
Past (n = 1466, 23.7%)	0.84 (0.70–0.99)*	0.82 (0.69–0.99)*	0.86 (0.71–1.04)	0.87 (0.72–1.04)
Current (n = 554, 8.9%)	0.86 (0.65–1.13)	0.81 (0.62–1.07)	0.84 (0.63–1.13)	0.91 (0.69–1.19)
**Experience of cat ownership**				
Never § (n = 5118, 82.6%)	1	1	1	1
Past (n = 693, 11.2%)	0.89 (0.70–1.12)	0.89 (0.70–1.13)	0.88 (0.68–1.14)	0.92 (0.73–1.16)
Current (n = 386, 6.2%)	1.04 (0.77–1.40)	1.04 (0.76–1.42)	1.02 (0.73–1.40)	1.08 (0.80–1.46)

^*^P < 0.05, **P < 0.01; ^§^Reference group. OR: odds ratio.

Mixed-effects logistic regression models were run separately. The random effects were the 18 administrative districts.

Multivariate-adjusted for sex, age, household size, equivalent income, history of stroke, food variety, Geriatric Depression Scale 5 score, alcohol status, and smoking status

Physical function was assessed by motor fitness scale and physical activity was exercise habit. Social function was assessed by interaction with neighbors.

In stratified analysis, mixed-effects logistic regression models yielded an OR of 0.81 (0.62–1.07) in current dog ownership and 0.82 (0.69–0.99) in past dog ownership for incident frailty, as compared with never owners of dogs, after controlling for important confounders. For cat ownership, current cat owners showed OR of 1.04 (0.76–1.42) and past cat owners showed OR of 0.89 (0.70–1.13), as compared with never cat owners.

Furthermore, we tested the hypothesis that experience of dog ownership may have a key role in maintaining physical and social function and thus might help lower the risk of incident frailty. When physical function (motor fitness scale) and physical activity (exercise habit) were added to the model as potential covariates, ORs were not changed from multivariate-adjusted model, however, 95% confidence interval were wide and there is no significance in the association of experience of dog/cat ownership with incident frailty. When social function (interaction with neighbors) was added to the model, the association of dog/cat ownership with incident frailty was attenuated (OR of current owners was 0.95 and OR of past owners was 0.88).

## Discussion

This prospective study of community-dwelling older Japanese showed that, as compared with respondents with no history of dog/cat ownership, those with past ownership had a lower risk and those with current owners had slightly lower risk of incident frailty, even after adjustment for important confounders. In particular, the risk of incident frailty was significantly lower for past dog owners than for cat owners. Our previous cross-sectional study reported that current dog or cat owner tend to be less frailty^16^.

Specific mechanism between dog/cat ownership and incident frailty remains unclear, because this study does not rule out the pet ownership effect completely (e.g., individuals who are already more physically active tend to have a pet). This study added new finding that past dog ownership reduced the risk of subsequent frailty and raises two mechanisms for this effect. First, as a result of dog walking, dog owners are more likely to have daily physical activity and higher physical function. A meta-analysis showed that dog owners walked more and were more physically active than non-dog owners^5^. Dall *et al*. reported that older dog owners spent 22 more minutes walking and walked 2,670 more steps per day than did non-dog owners. This additional activity satisfies guidelines calling for 150 minutes of moderate physical activity per week^13^. In the present data, past dog/cat owners showed the same high motor fitness scale as current owners. Second, dog ownership might maintain higher social function. Wood *et al*. suggested that pets affect broader social interactions and perceptions, sense of community, and social capital at the neighborhood level^14^. In this study, we confirmed higher interaction with neighbors, such as significant relationship in past dog/cat owners than current and never owners. Dog owners might have more opportunities to extend their social network by chatting with neighbors while dog walking. Unfortunately, data on years of dog/cat ownership were not available in the present study, past owners may have longer years of ownership. These findings suggest that longer experience of dog ownership may enhance both of physical and social function for owners.

We used models to investigate the association of experience of dog ownership with incident frailty, after controlling for physical function, physical activity, and social function. The association of experience of dog ownership with incident frailty was attenuated when those functions were included in the statistical model, which supports the study hypothesis. Caring for a dog likely increases the owner’s physical activity and social network and may thus have an important role in maintaining physical and social function in later life. Higher physical and social function through dog ownership might help reduce subsequent frailty risk among older adults.

This study has strengths that warrant mention. First, our large sample of community-dwelling older Japanese revealed an association of experience of dog/cat ownership with frailty in the general population. The present study is a worthful addition to the field of human–animal interaction, as it enrolled a large sample of community-dwelling older adults, which enabled subgroup analysis of experience of dog and cat ownership. Second, important covariates were included in the analysis, thus enabling analysis of the independent association of experience of dog/cat ownership with incident frailty after controlling for important sociodemographic and health characteristics.

Nevertheless, this study has limitations. First, although data on years of dog/cat ownership and frequency of dog walking were important factor to explore the mechanisms between experience of ownership and frailty, these data were not available for analysis. Future study should compare total years of ownership between current and past owners. Second, previous studies reported several health benefits associated with cat ownership^14,15^; however, the present study showed no positive association between experience of cat ownership and incident frailty. The reason for this absence of an effect is unknown, and we hope that future studies will examine associations between cat ownership and frailty in different countries, such as Western populations. Third, we use the data from older adults to consider disadvantages through their pats, specific information such as pet loss^11^, allergy, and zoonosis were not included. Future studies should confirm the association of dog ownership with frailty among older adults who had experienced negative effects by pet ownership.

This is the first study to identify an association between experience of dog ownership and incident frailty even after adjustment for important sociodemographic and health characteristics among community-dwelling older adults. Longer experience of caring for a dog likely increases physical activity and time spent outdoors and may therefore have an important role in maintaining physical and social function and reducing frailty risk among older adults. These findings should be helpful in developing health promotion strategies for preventing and reducing frailty among older adults.

## Method

### Participants

Data for this study were collected as part of a community-wide intervention trial (the Ota Genki Senior Project) in 18 administrative districts of Ota City, Tokyo^27^. Ota City is the southernmost of the 23 special wards of Tokyo. The population was 716,645 and 162,443 were aged 65 years or older on August in 2016. The proportion of elders to the total population was 22.7%^27^. In 2016, we mailed a self-administered questionnaire to 15,500 older residents as a baseline survey; 11,925 questionnaires were returned (response rate 76.9%). In 2018, we conducted a mail survey in the same area (response rate 74.2%) and collected 10,837 questionnaires. Mean age and percentage of female were almost the same between responders in 2016 and 2018. To be eligible for the study, individuals had to be non-frail and to complete a questionnaire on their experience of dog/cat ownership in 2016 (n = 7,881). Ultimately, data from 6,197 non-frail community-dwelling adults were followed in 2018. Among participants who were included the study (n = 6,197) and excluded from loss of follow-up (n = 1,684), the differences of dog/cat ownership, sex and age were not significant. All methods according to the Ota Genki Senior Project were carried out in accordance with the relevant guidelines of the Ethical Committee of the Tokyo Metropolitan Institute of Gerontology. We adhered strictly to the Declaration of Helsinki. This study was approved by the Ethical Committee of the Tokyo Metropolitan Institute of Gerontology. A statement attached to the questionnaire explained the purpose of the survey, the voluntary nature of participation, and a promise of anonymity in the analysis. Returning the questionnaire was considered as consent to participate in the study.

### Dog/cat ownership

Participants were asked about their experience living with a pet (current, past, or never). Those with current or past pet experience were asked about pet species in the household (dog, cat, or other).

### Incident frailty

Frailty was defined as a score of four or higher on the Kaigo-Yobo Check-List 15 (CL15, Supplementary information), which had concurrent and predictive validity, and good reliability as a questionnaire-based scale for screening high-risk Japanese older adults^28,29^. Kojima *et al*. compared CL15 to Frailty Index in predicting risks of long-term care insurance certification and/or mortality over 3 years^30^. Although CL15 includes smaller numbers of items than Frailty Index, CL15 was shown to be highly correlated with Frailty Index, significant predictors of long-term care insurance certification and/or mortality, and compatible to Frailty Index in the risk prediction^30^.

### Other variables

The covariates included frailty-associated sociodemographic and health characteristics, namely, sex, age, household size, marital status, educational attainment, equivalent income, history of chronic diseases, history of hospitalization during the past year, fall during the past year, alcohol drinking, smoking status, food variety, body mass index, exercise habit, interaction with neighbors, frequency of going outdoors, self-rated health, and Geriatric Depression Scale (GDS)-5 score.

The chronic diseases evaluated included clinically relevant medical conditions, namely, hypertension, hyperlipidemia, heart disease, stroke, diabetes mellitus, and lung respiratory disease. For each of these conditions, participants were asked if they had received a physician diagnosis (yes or no)^31,32^. Food variety was assessed by a dietary variety score, which was calculated by using the consumption frequencies for 10 food items (meat, fish/shellfish, eggs, milk, soybean products, green/yellow vegetables, potatoes, fruit, seaweed, and fats/oils) during the week. The score ranges from 0 to 10, and higher scores indicate greater food variety^33^. Participants were classified as having a daily exercise habit if they reported one or more type of daily exercise (i.e., walking, running, weight training, gymnastic exercises, swimming, cycling, yoga, or others). Interaction with neighbors was classified as close relationship, conversation level, exchange of greetings only, and no social contact. GDS-5 is a screening tool to identify symptoms of depression in the older population^34^ and higher score indicates the presence of depressive symptoms.

### Statistical analyses

First, associations of baseline demographic and comprehensive health characteristics with experience of dog/cat ownership were tested by using the chi-square test or t-test. Second, we used mixed-effects logistic regression models to examine independent associations of experience of dog/cat ownership with incident frailty, after controlling for potential confounders. The random effects were the study area. Potential confounders were evaluated for collinearity and chosen; sex, age, household size, equivalent income, history of stroke, food variety, GDS-5, alcohol status, and smoking status. In addition, using mixed-effects logistic regression models after controlling for important confounders, we conducted stratified analysis for experience of dog/cat ownership. Statistical analyses were conducted with SPSS (version 23.0; IBM Corp, Armonk, NY, USA) and SAS (version 9.4; SAS Institute, Inc., Cary, NC, USA). A *P-*value of less than 0.05 was considered to indicate statistical significance.

## Supplementary information

Assessment for frailty in this study:The Kaigo-Yobo Check List 15 (CL15)

## Data Availability

The data of a community-wide intervention trial (Ota Genki Senior Project) in Ota City contains sensitive participant information and cannot be released publicly due to ethicolegal restrictions imposed by the Ethics Committee at Tokyo Metropolitan Institute of Gerontology. The datasets generated during and/or analysed during the current study are available from the corresponding author on reasonable request. Long-term storaged data is available upon reasonable request to Yu Taniguchi (yu0717@tmig.or.jp) and Satoshi Seino (seino@tmig.or.jp).

## References

[1] Bergroth E (2012). Respiratory tract illnesses during the first year of life: effect of dog and cat contacts. Pediatrics.

[2] Owen CG (2010). Family dog ownership and levels of physical activity in childhood: findings from the Child Heart and Health Study in England. American journal of public health.

[3] Heyworth JS, Cutt H, Glonek G (2006). Does dog or cat ownership lead to increased gastroenteritis in young children in South Australia?. Epidemiology and infection.

[4] Coleman KJ (2008). Physical activity, weight status, and neighborhood characteristics of dog walkers. Preventive medicine.

[5] Christian HE (2013). Dog ownership and physical activity: a review of the evidence. Journal of physical activity & health.

[6] Headey B, Na NF, Zheng. RP (2008). Dogs Benefit Owners’ Health: A ‘Natural Experiment’ in China. Social Indicators Research.

[7] Mubanga M (2017). Dog ownership and the risk of cardiovascular disease and death - a nationwide cohort study. Scientific reports.

[8] Qureshi AI, Memon MZ, Vazquez G, Suri MF (2009). Cat ownership and the Risk of Fatal Cardiovascular Diseases. Results from the Second National Health and Nutrition Examination Study Mortality Follow-up Study. Journal of vascular and interventional neurology.

[9] Allen K, Shykoff BE, Izzo JL (2001). Pet ownership, but not ace inhibitor therapy, blunts home blood pressure responses to mental stress. Hypertension (Dallas, Tex.: 1979).

[10] Siegel JM, Angulo FJ, Detels R, Wesch J, Mullen A (1999). AIDS diagnosis and depression in the Multicenter AIDS Cohort Study: the ameliorating impact of pet ownership. AIDS care.

[11] Mueller MK, Gee NR, Bures RM (2018). Human-animal interaction as a social determinant of health: descriptive findings from the health and retirement study. BMC public health.

[12] Thorpe RJ (2006). Dog ownership, walking behavior, and maintained mobility in late life. J Am Geriatr Soc.

[13] Dall PM (2017). The influence of dog ownership on objective measures of free-living physical activity and sedentary behaviour in community-dwelling older adults: a longitudinal case-controlled study. BMC public health.

[14] Wood LJ, Billie G, Max K, AB. D (2007). More Than a Furry Companion: The Ripple Effect of Companion Animals on Neighborhood Interactions and Sense of Community. Society & Animals.

[15] Turner DC, Rieger G, Gygax L (2003). Spouses and cats and their effects on human mood. *A Multidisciplinary*. Journal of The Interactions of People & Animals.

[16] Taniguchi Y (2018). Physical, social, and psychological characteristics of community-dwelling elderly Japanese dog and cat owners. PloS one.

[17] Clegg A, Young J, Iliffe S, Rikkert MO, Rockwood K (2013). Frailty in elderly people. Lancet (London, England).

[18] Kojima G, Taniguchi Y, Iliffe S, Walters K (2016). Frailty as a Predictor of Alzheimer Disease, Vascular Dementia, and All Dementia Among Community-Dwelling Older People: A Systematic Review and Meta-Analysis. Journal of the American Medical Directors Association.

[19] Kojima G (2015). Frailty as a Predictor of Future Falls Among Community-Dwelling Older People: A Systematic Review and Meta-Analysis. Journal of the American Medical Directors Association.

[20] Kojima G (2016). Frailty as a predictor of fractures among community-dwelling older people: A systematic review and meta-analysis. Bone.

[21] Kojima G (2017). Frailty as a predictor of disabilities among community-dwelling older people: a systematic review and meta-analysis. Disability and rehabilitation.

[22] Kojima G (2016). Frailty as a predictor of hospitalisation among community-dwelling older people: a systematic review and meta-analysis. Journal of epidemiology and community health.

[23] Kojima G (2015). Prevalence of Frailty in Nursing Homes: A Systematic Review and Meta-Analysis. Journal of the American Medical Directors Association.

[24] Kojima G (2018). Frailty Defined by FRAIL Scale as a Predictor of Mortality: A Systematic Review and Meta-analysis. Journal of the American Medical Directors Association.

[25] Kojima G, Iliffe S, Walters K (2018). Frailty index as a predictor of mortality: a systematic review and meta-analysis. Age Ageing.

[26] Makizako Hyuma, Shimada Hiroyuki, Doi Takehiko, Tsutsumimoto Kota, Hotta Ryo, Nakakubo Sho, Makino Keitaro, Lee Sangyoon (2018). Social Frailty Leads to the Development of Physical Frailty among Physically Non-Frail Adults: A Four-Year Follow-Up Longitudinal Cohort Study. International Journal of Environmental Research and Public Health.

[27] Seino, S. *et al*. A community-wide intervention trial for preventing and reducing frailty among older adults living in metropolitan areas: Design and baseline survey for a study integrating participatory action research with cluster trial. *J Epidemiol* In press (2018). 10.2188/jea.JE20170109 PMC6336723 29962492

[28] Shinkai S (2010). Research on screening for frailty: development of “the Kaigo-Yobo Checklist”. [Nihon koshu eisei zasshi] Japanese journal of public health.

[29] Shinkai S (2016). Public health approach to preventing frailty in the community and its effect on healthy aging in Japan. Geriatrics & gerontology international.

[30] Kojima Gotaro, Taniguchi Yu, Kitamura Akihiko, Shinkai Shoji (2018). Are the Kihon Checklist and the Kaigo-Yobo Checklist Compatible With the Frailty Index?. Journal of the American Medical Directors Association.

[31] Taniguchi Y, Yoshida H, Fujiwara Y, Motohashi Y, Shinkai S (2012). A prospective study of gait performance and subsequent cognitive decline in a general population of older Japanese. J Gerontol A Biol Sci Med Sci.

[32] Taniguchi Y (2014). Nutritional biomarkers and subsequent cognitive decline among community-dwelling older Japanese: a prospective study. J Gerontol A Biol Sci Med Sci.

[33] Kumagai S (2003). Effects of dietary variety on declines in high-level functional capacity in elderly people living in a community. Nihon koshu eisei zasshi] Japanese journal of public health.

[34] Hoyl MT (1999). Development and testing of a five-item version of the Geriatric Depression Scale. Journal of the American Geriatrics Society.

