# Supplementary material for: Association of Dog and Cat Ownership with Incident Frailty among Community-Dwelling Elderly Japanese
Source: Sci Rep. 2019 Dec 9;9:18604. doi: 10.1038/s41598-019-54955-9 (PMC6901519; doi:10.1038/s41598-019-54955-9)
Supplement: Supplementary file 1 — Assessment for frailty in this study:The Kaigo-Yobo Check List 15 (CL15) [file 41598_2019_54955_MOESM1_ESM.pdf]

Association of Dog and Cat Ownership with Incident Frailty among Community-Dwelling Elderly Japanese

Yu Taniguchi [1*], Satoshi Seino [1], Mariko Nishi [1], Yui Tomine [1], Izumi Tanaka [1], Yuri Yokoyama [1], Tomoko Ikeuchi [1], Akihiko Kitamura[1], Shoji Shinkai [1]

1 Research Team for Social Participation and Community Health, Tokyo Metropolitan Institute of Gerontology, Tokyo, Japan

Address for correspondence:

*Yu Taniguchi, PhD

Research Team for Social Participation and Community Health,

Tokyo Metropolitan Institute of Gerontology,

35-2 Sakae-cho, Itabashi-ku, Tokyo 173-0015, Japan.

E-mail: yu0717@tmig.or.jp

TEL: +81(3)3964-3241 ext. 4252; FAX: +81(3)3964-4776

Appendix. Assessment for frailty in this study: the Kaigo-Yobo Check-List 15 (CL15).

Do you usually stay at home all day long? (Yes/No)

How often do you usually go out? (More than once per 2-3 days, Less than once a week)

Do you have any hobby? (Yes/No)

Do you have neighbors who you can talk closely with? (Yes/No)

Besides your neighbors, do you have close friends, families, or relatives who you visit? (Yes/No)

Have you experienced a fall in the past year? (Yes/No)

Can you walk for 1 km? (Yes/No)

Can you see things clearly? (Without difficulty, With difficulty or cannot)

Do you often slip or stumble at home? (Yes/No)

Do you refrain from going out because of fear of falling? (Yes/No)

Have you been hospitalized in the past year? (Yes/No)

Do you have appetite these days? (Yes/No)

Do you have any difficulty chewing? (Yes/No)

Have you lost 3 kg or more in the past 6 months? (Yes/No)

Do you think you have lost muscle or fat in the past 6 months? (Yes/No)
